# Intraosseous Hemangioma of the Inferior Turbinate

**DOI:** 10.1155/2010/409429

**Published:** 2010-03-15

**Authors:** Kazuya Takeda, Yukinori Takenaka, Michiko Hashimoto

**Affiliations:** Department of Otolaryngology, Kinki Central Hospital, 3-1 Kurumazuka, Itami, Hyogo 664-8533, Japan

## Abstract

The nasal cavity harbors an enormous variety of neoplasms, including epithelial and mesenchymal tumors. Hemangioma is an infrequent mesenchymal tumor of the nasal cavity, mostly arising in the mucosa and rarely in the bones. We describe the case of a 73-year-old woman who was referred to our hospital with a tumor in her left nasal cavity. The tumor originated from the left inferior turbinate. Histological examination subsequent to complete excision revealed that the tumor was an intraosseous cavernous hemangioma. To our knowledge, this is the second case of intraosseous hemangioma of the inferior turbinate reported in the English literature.

## 1. Introduction

A wide variety of tumors occur in the nasal cavity. Hemangiomas account for about 20% of all benign neoplasms of the nasal cavity. Hemangioma of the nasal cavity occurs most commonly on the septum (65%), lateral wall (18%), and vestibule (16%) [1]. Nasal hemangiomas mostly arise from the soft tissues of the nasal cavity. Although intranasal hemangiomas sometimes cause bony changes or destruct nasal bones, they rarely arise from these bones. 

Hemangiomas also occur as solitary lesions in bones. These tumors account for only 0.7% of all primary bone tumors [2]. Intraosseous hemangiomas usually occur in the vertebral column and skull bones. Intraosseous hemangioma of the nasal cavity is extremely rare; only one case has been reported in the English literature [3]. Here we report a case of intraosseous hemangioma of the inferior turbinate. 

## 2. Case Report

**Figure 1:**
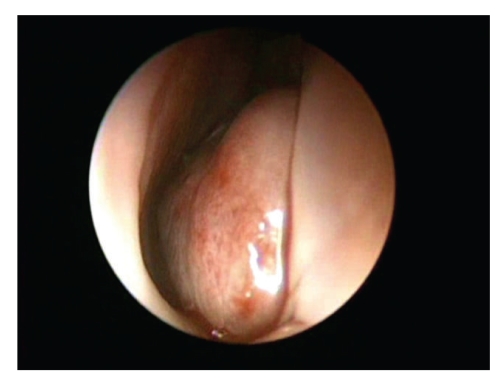
Fiberscopic view showing the mass arising from the left inferior turbinate.

**Figure 2:**
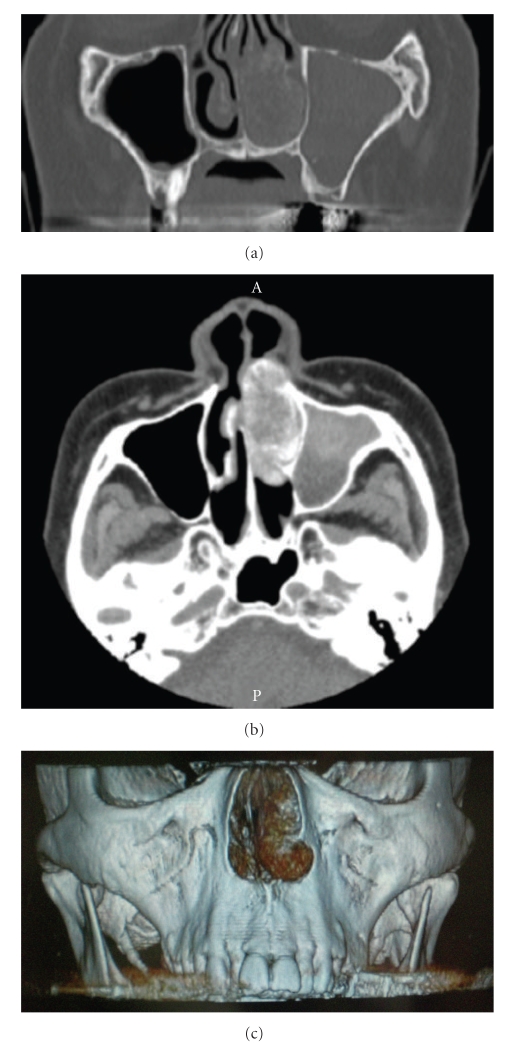
Computed tomography ((a): coronal section, (b): axial section and (c): three-dimensional reconstruction) showing the mass of the inferior turbinate that filled the nasal cavity.

A 73-year-old woman was referred to our department with a 1-month history of left-sided nasal obstruction. She had no history of epistaxis or facial trauma. Anterior rhinoscopic examination revealed a mass obstructing the left nasal cavity. A hypertrophied inferior turbinate seemed to be occupying the left nasal cavity. The tumor was bony hard and covered with intact mucosa not hypervascularized mucosa (Figure 1). No other specific findings were observed in the head and neck lesions. Unenhanced computed tomography showed that the bony tumor replaced the anterior portion of the left inferior turbinate. It had a characteristic appearance of intraosseous hemangioma, known as a honeycomb or sunburst appearance. Neither erosion nor destruction of surrounded tissues was observed. Deviation of the nasal septum and opacification of the left maxillary sinus were observed (Figure 2). 

Under general anesthesia, the patient underwent surgical excision by the Caldwell-Luc procedure. The tumor, inferior turbinate, and medial wall of the maxillary sinus were resected en bloc. Intraoperative hemorrhage was 20 mL. The nasal cavity was packed with gauze. The packing was removed on the fifth postoperative day. The postoperative course was uneventful, and there was no evidence of recurrence at 8-month follow-up. 

**Figure 3:**
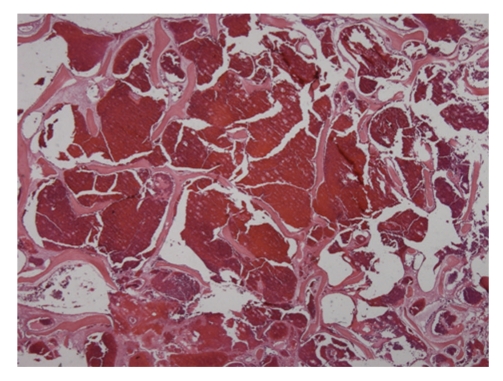
Histological examination (hematoxylin-eosin stain) showing the tumor composed of blood-filled, thin-walled vessels between the bony trabeculae. The lesion was diagnosed as intraosseous hemangioma of the inferior turbinate.

Macroscopically, the tumor was 4 × 5 cm in size and covered with intact mucosa. Microscopically, the tumor composed of bony trabeculae and anastomosing vascular channels of cavernous size. The histological diagnosis was cavernous hemangioma (Figure 3). 

## 3. Discussion

Hemangiomas are benign tumors originating in the vascular tissues of skin, mucosa, muscles, glands, and bones. Although head and neck lesions are common sites for hemangioma, hemangiomas of the nasal cavity are rare. The most common site for nasal hemangiomas is the nasal septum, followed by the lateral wall and vestibule [1]. Several reports have shown a hemangioma arising in the turbinate [4–7]. However, most of them arise from the mucosa. 

Hemangiomas occur not only in soft tissues but also in bones. Intraosseous hemangiomas account for only 0.7% of all primary bone tumors. The most common sites in the head and neck are the skull (53%), mandible (10.7%), nasal bones (9%), and cervical vertebrae (6%). 

Intraosseous hemangiomas originating in the nasal cavity are extremely rare. Only one case of hemangioma within the turbinate bone has been reported in the English literature [3]. 

The cause of intraosseous hemangioma is not well understood. Although many patients have a history of local trauma, a causal relationship remains doubtful [8]. In our case, there was no history of facial trauma. The lesions occur twice as often in females as in males. In contrast to soft tissue hemangiomas, which are most common in children, osseous hemangiomas are more common in older populations [9]. 

Diagnosis of intraosseous hemangioma is extremely difficult. It presents as a slowly enlarging, hard mass. It usually does not present signs that suggest a vascular lesion (e.g., bluish purple discoloration, spontaneous hemorrhage) [9]. Radiographic examination is helpful in diagnosing intraosseous hemangiomas because these tumors have a characteristic appearance [9], that is, a discrete honeycombed area created by multiple cavernous spaces in the lesion, sunburst pattern of radiating trabeculations, and soap-bubble appearance. Other imaging techniques have been used in the diagnosis. Angiography typically shows increased vascularity in the area of the tumor, with feeder vessels but no large draining veins. 

Based on histopathological examination, hemangiomas can be subdivided into two types, that is, capillary and cavernous types. Although cavernous hemangiomas of the nasal cavity are uncommon, most intraosseous hemangiomas show a cavernous pattern [9, 10]. 

Therapeutic approaches of intraosseous hemangioma include surgery, radiotherapy, sclerotherapy, and embolization [2, 9, 11, 12]. Although hemangiomas are responsive to radiotherapy, long-term side effects, such as malignancy, region growth impairment, and scarring, render it an unfavorable treatment modality. Therefore, radiotherapy is reserved for unresectable lesions [8]. Some authors advocated transarterial embolization and sclerotherapy; however, these procedures are palliative [2]. Complete surgical excision with or without preoperative embolization is the mainstay of treatment [8, 11, 13]. Partial resection may be a treatment of choice because complete tumor resection sometimes requires a long incision and reconstruction with bone grafts or alloplastic implants [9]. 

Although radiological diagnosis of intraosseous hemangioma has been established, clinical and computed tomographic evidence does not always lead to an exact diagnosis. Therefore, surgery should play a definite role in both diagnosis and treatment. 

In summary, we report a case of intraosseous hemangioma of the inferior turbinate. Intraosseous hemangiomas in unusual sites pose diagnostic difficulties. The possibility of intraosseous hemangioma must be considered when a bony mass is detected in the nasal cavity.
